# High stability of the genome of *Akkermansia muciniphila* Muc^T^ under long-term culturing conditions

**DOI:** 10.1128/spectrum.02400-25

**Published:** 2026-03-10

**Authors:** Kate Ligthart, Janneke Elzinga, Anneleen Segers, Hauke Smidt, Willem M. de Vos

**Affiliations:** 1 Laboratory of Microbiology, Wageningen University & Research, Wageningen, the Netherlands; 2 Human Microbiome Research Program, Faculty of Medicine, University of Helsinki, Helsinki, Finland; Griffith University-Gold Coast Campus, Gold Coast, Queensland, Australia

**Keywords:** *Akkermansia muciniphila*, genome stability, phase variation, mucus binding

## Abstract

To advance our understanding of the genomic stability of *Akkermansia muciniphila*, its type strain Muc^T^ was grown for over 1,000 generations at five different culturing conditions, followed by the isolation of single colonies and their subsequent genomic DNA sequencing, physiological, and functional analysis. Notably, not a single mutation was found in the genomes using our sequencing approach of the dozen strains isolated after growth for 1,000 generations in media containing porcine gastric mucin, either grown with or without shaking. Similarly, strains isolated from cultures grown in minimal medium containing high or low N-acetylglucosamine, or low N-acetylglucosamine containing 1% ox bile, exhibited very low mutation rates between 2.1 × 10^−10^ and 8.6 × 10^−11^ per nucleotide per generation. From these, a total of five unique strains were obtained that contained one or more nucleotide variations. Subsequent analysis and characterization revealed that two of the five strains included a G duplication in a nonanucleotide homopolymer G region in the gene with the locus tag Amuc_1413, resulting in a frameshift and a subsequent loss of mucin binding capacity. Analysis of published genomes of *Akkermansia* spp. confirmed the instability of this nonanucleotide G region in this Amuc_1413 gene, predicted to be involved in exopolysaccharide export. These findings provide valuable insight into the stability of the *A. muciniphila* genome and identify phase variation as a mechanism that can explain some of the earlier reported heterogeneity. We conclude that *A. muciniphila* Muc^T^ has large genomic stability under long-term culturing conditions and identified the Amuc_1413 protein as essential for mucus binding.

**IMPORTANCE:**

*Akkermansia muciniphila* Muc^T^ has emerged as a next-generation beneficial microbe due to its capacity to improve gut barrier function in mouse models and humans. To assess the potential of *A. muciniphila* Muc^T^ for industrial applications, we studied the genomic stability by cultivating different growth conditions for over 1,000 generations. We found that the genome of *A. muciniphila* Muc^T^ is highly stable when grown on mucin medium and relatively stable when grown in industrial media. Additionally, we characterized the obtained mutants that identified phase variation as a mechanism operating in *A. muciniphila*, which allowed us to identify the gene with the locus tag Amuc_1413, encoding a protein involved in exopolysaccharide production, to be involved in mucus binding.

## INTRODUCTION

*Akkermansia muciniphila* strain Muc^T^ is a mucin-degrading symbiont found in the intestine of humans and other animals that has become of particular interest due to its potential beneficial effect on host health (1, 2). Its presence in the human gut is inversely correlated with low microbial gene richness (3) and several diseases such as obesity and type 2 diabetes (4, 5). Administration of *A. muciniphila* Muc^T^ was found to reduce high-fat diet-induced metabolic disorders in mice (6). Similar results were observed with pasteurized *A. muciniphila* Muc^T^, suggesting that metabolic activity is not causing this effect (7). The likely key component of the mode of action is Amuc_1100, a membrane-associated, pili-associated signaling protein (7, 8). The Amuc_1100 protein activates Toll-like receptor 2 (TLR2), is thermostable, and thus unaffected by pasteurization (7). Since its discovery, the Amuc_1100 protein has been found to improve gut-barrier function, reduce intestinal inflammation, as well as improve obesity-related symptoms in mice (7–10). A recent study used transposon mutagenesis to identify a set of genes essential for mucin degradation and uptake, which were also crucial for effective colonization of the mouse gut by *A. muciniphila* (11). These findings underscore the complex interaction between *A. muciniphila* and host physiology, particularly regarding the mucus layer.

Several studies in humans have found a negative correlation between the abundance of *A. muciniphila* and conditions such as obesity, type 2 diabetes, and hypertension (1, 4, 6). However, it is important to note that genome-based phylogenetic analyses have revealed the existence of several distinct species-like taxa within *A. muciniphila* designated AmIa, AmIb, AmII, AmIII, and AmIV (12, 13). In spite of the fact that all share a highly similar 16S rRNA sequence with *A. muciniphila*, a new nomenclature has been proposed for several of these, including *A. massiliensis* (AmII), *A. biwaensis* (AmIV), *A. ignis* (AmV), and *A. durhamii* (AmVI) (14). Of note, these species-like taxa were found to be correlated with different health outcomes, with a reduced risk for type 2 diabetes and low body mass index specifically linked to AmI, which includes the type strain *A. muciniphila* Muc^T^ (12, 14).

A human trial with *A. muciniphila* Muc^T^ has also shown that administering pasteurized *A. muciniphila* Muc^T^ is safe, improved insulin sensitivity, and slightly decreased body weight, fat mass, and hip circumference (15). This has been supported by the toxicological analysis of pasteurized *A. muciniphila* Muc^T^ (16) that stood at the basis to consider products with this strain safe for human consumption in Europe and self-affirmed GRAS in the USA (17). Hence, *A. muciniphila* Muc^T^ is a next-generation beneficial microbe, and its pasteurized cells are on the market worldwide. In this context, it is important to know whether the genome of *A. muciniphila* Muc^T^ remains stable over successive generations of growth in laboratory and industrial manufacturing conditions, as stability of bacterial genomes during the manufacturing process is an important factor influencing the feasibility of utilizing potential beneficial strains in industrial applications (18, 19).

Bacterial properties can change over time due to the accumulation of small mutations such as single nucleotide variations, insertions, or deletions in their genomes. To determine the stability of a single strain’s genome, experimental evolution studies can be performed. In these studies, bacteria are grown for many generations, often under multiple conditions, followed by the analysis of the genomes of single isolates. This was most famously done for *Escherichia coli*, which was first grown for 2,000 generations in Davis minimal broth, supplemented with thiamine hydrochloride and glucose. The survivors exhibited significantly higher fitness than the starting strain, primarily achieved in the first 1,000 generations (20). After continuing this experiment for over 40,000 generations, the genomes of the *E. coli* isolates were sequenced at different generations. This revealed a constant mutation rate of around 1.6 × 10^–10^ per nucleotide per generation up until generation 20,000 (21).

To advance our understanding of the stability of its genome, we grew *A. muciniphila* Muc^T^ for 1,000 generations in five different conditions. These conditions include a control medium, the same medium that was used to initially discover and characterize *A. muciniphila* Muc^T^, containing mucin (2). Next, the same medium was used with continuous shaking to cause shear stress as a model for industrial conditions. In addition, a synthetic minimal medium (MM) was used with glucose (Glc) and N-acetylglucosamine (GlcNAc) in an equimolar ratio replacing mucin. This medium has been developed for large-scale industrial growth conditions and is also frequently used in lab settings, as mucin is not a food-grade component and can interfere with downstream processing (22, 23). We also tested this MM with an increased Glc:GlcNAc ratio of 10:1, which may affect gene expression in some conditions (22). Finally, we included MM with Glc:GlcNAc in a 10:1 ratio with added ox bile as a stressor, known to inhibit growth and alter gene expression (24). By transferring exponentially growing cultures to fresh medium daily, we selected for the fastest growers on each medium. These were finally studied after 1,000 generations of growth by isolating and characterizing single colony isolates by high-throughput DNA sequence analysis and phenotypic characterization. Through these experiments, we aimed to explore the stability of the *A. muciniphila* Muc^T^ genome, as well as characterize the impact of the potentially mutated genes. The results indicate a high genomic stability of *A. muciniphila* Muc^T^ under mucus growth and industry-like conditions. A detailed analysis of obtained mutants allowed us to identify a nonanucleotide homopolymeric G region that shows features of phase variation and is located in a gene that is involved in mucus binding.

## RESULTS

**Fig 1:**
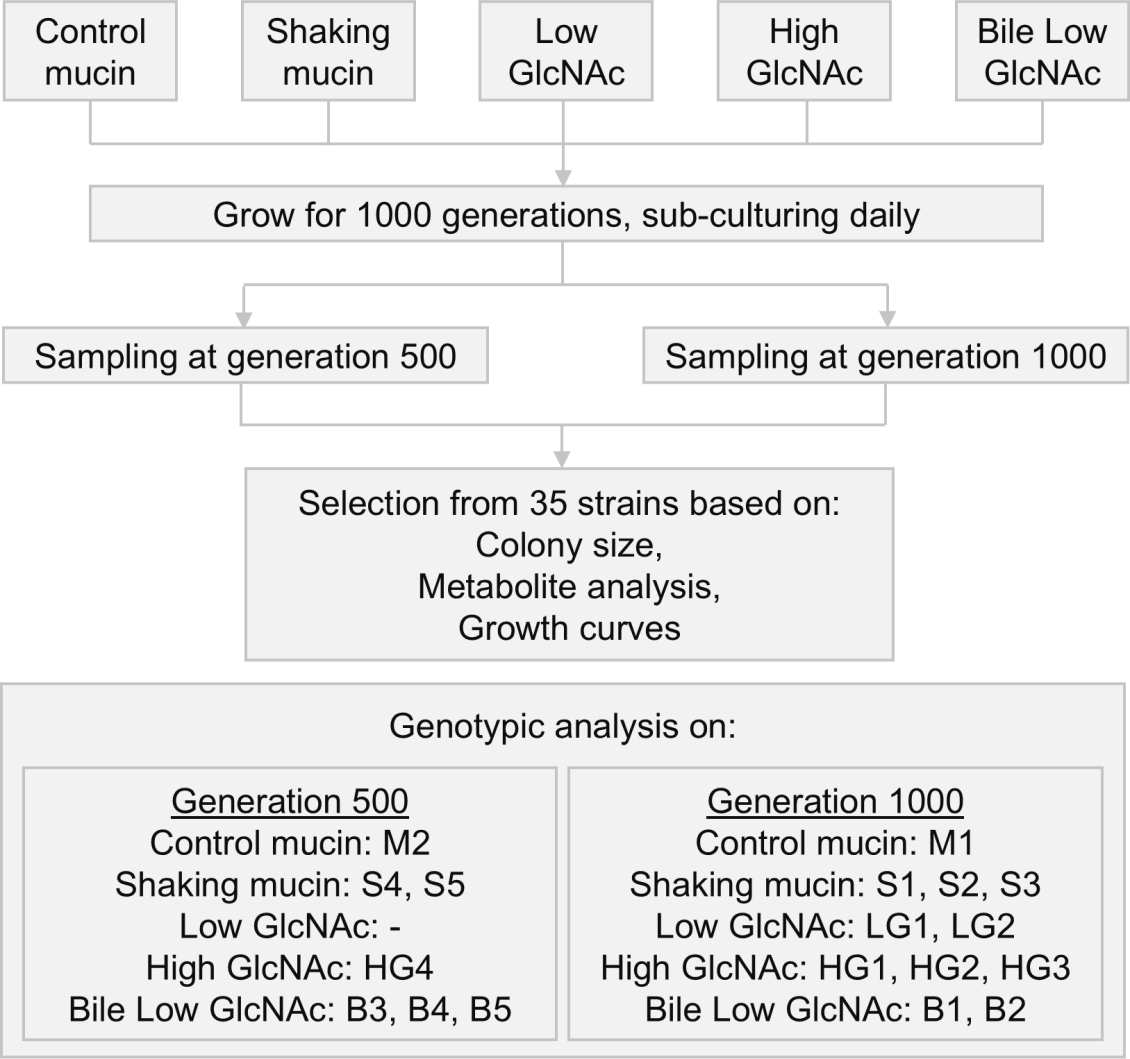
Workflow. *A. muciniphila* Muc^T^ was grown for 1,000 generations in five different conditions. Samples were taken at generation 500 and 1,000, and the subsequent selection for genotypic analysis was based on colony size, metabolite analysis, and growth curves.

We cultured the founder strain *A. muciniphila* Muc^T^ for 1,000 generations in the following five distinct conditions (Fig. 1): (i) Control Mucin, consisting of MM with porcine gastric mucin; (ii) Shaking Mucin, which used the same medium as Control Mucin but with continuous shaking at 200 rpm; (iii) Low GlcNAc, with a 10:1 molar ratio of Glc:GlcNAc in MM; (iv) High GlcNAc, with an equimolar ratio of Glc:GlcNAc in MM; and (v) Bile Low GlcNAc, which used the same medium as the Low GlcNAc condition but with the addition of ox bile (2, 23).

At generation 500 and 1,000, we collected and plated samples from each of the five conditions. From these, we selected seven colonies per condition, ensuring to select both larger and smaller colonies. In this way, a total of 35 strains were obtained. Preliminary phenotypic screening was performed and included colony size, growth rate, and metabolite consumption to identify which strains to use for further genomic analysis (Fig. 1).

Based on these initial phenotypic analyses, where we noted some growth differences, we selected 18 strains for genotypic analysis by draft genome sequencing and further phenotypic characterization (Fig. 1). From the Control Mucin condition, we chose M1 (generation 1,000) and M2 (generation 500). For the Shaking Mucin condition, we selected S1, S2, and S3 (generation 1,000) and S4 and S5 (generation 500). From the Low GlcNAc condition, LG1 and LG2 (generation 1,000) were chosen. From the High GlcNAc condition, HG1, HG2, and HG3 (generation 1,000) and HG4 (generation 500) were selected. Finally, from the Bile Low GlcNAc condition, we chose B1 and B2 (generation 1,000) and B3, B4, and B5 (generation 500) (Table 1).

**TABLE 1:** Overview of sequenced strains

Growth condition	Strain	Generation	No. of mutations	Mutations/nt/gen
–	Muc^T^	0	0^*a*^	–^*b*^
Control Mucin	M1	1,000	0	–
	M2	500	0	
Shaking Mucin	S1	1,000	0	–
	S2	1,000	0	
	S3	1,000	0	
	S4	500	0	
	S5	500	0	
Low GlcNAc	LG1	1,000	3	2.14 × 10^−10^
	LG2	1,000	3	
High GlcNAc	HG1	1,000	2	5.63 × 10^−10^
	HG2	1,000	2	
	HG3	1,000	3	
	HG4	500	1	
Bile Low GlcNAc	B1	1,000	2	8.58 × 10^−11^
	B2	1,000	1	
	B3	500	0	
	B4	500	1	
	B5	500	0	

^*a*^ The two deletions identified in the founder strain *A. muciniphila* Muc^T^ were present in all sequenced strains (for explanation, see text).

^*b*^ –, no mutation.

While subsequent and repeated phenotypic testing did not reveal reproducible and significant growth differences, further genomic characterization showed unexpected insight into the number and nature of accumulated mutations, as discussed below. A notable observation was that the founder strain Muc^T^ contained two variations compared to the published *A. muciniphila* Muc^T^ reference genome (NCBI RefSeq: GCF_000020225.1, Table 1). One is a deletion of a single G nucleotide at position 1704819, located in the *recG* gene Amuc_1422. This mutation was also previously observed in *A. muciniphila* Muc^T^ obtained from the ATCC culture collection (11). Our founder strain Muc^T^ originated from the Wageningen Microbiology Strain Collection, the laboratory where the *A. muciniphila* type strain was first isolated (2). Hence, we conclude that there is indeed an error in the genome of *A. muciniphila* Muc^T^, which was obtained from the Wageningen laboratory and sequenced using Sanger and 454 pyrosequencing technologies prior to its publication in 2011 (11, 25). The other variation is a deletion of the following 43 nucleotides starting at nt position 2008639: 5′-TGCCCAATTAATATCTGATATTTTAGAATTAATATGGTCGTA-3′. This deletion is located in the coding sequence of Amuc_1661, resulting in a truncation of the predicted 261-residue hypothetical protein that has not been found to be expressed in proteome data sets (26). We consider this variation to be of minor significance and likely a consequence of initial repeated subculturing.

### Mutation analysis by genome sequencing

A total of 18 strains obtained after 500 or 1,000 generations of growth (Table 1) were analyzed at the genomic level by Illumina sequencing, together with the founder strain Muc^T^. The genome-analysis programs Geneious and PATRIC were applied to detect genomic variations when compared to the earlier published genome of *A. muciniphila* Muc^T^ (25). The results of both analyses were identical and revealed the same variations in the genomes in 9 of the 18 strains (Table 2; File S4).

**TABLE 2:** Overview of mutations found in sequenced strains^*a*^

	Mutation III			Mutation II			Mutation III		
Strain(s)	Location	Nt change	AA change	Location	Nt change	AA change	Location	Nt change	AA change
Muc^T^	Amuc_1422	nt1704819DelG		Amuc_1661	nt2008639 del 43-bp				
LG1/2	Amuc_1041	549_551DelCCG	Arg184Del	Amuc_1412	401 A->C	Gln134Pro	Amuc_1413	163DupG	55Frameshift
HG1/2	Amuc_1412	401 A->C	Gln134Pro	Amuc_1413	163DupG	55Frameshift			
HG3	Amuc_1412	401 A->C	Gln134Pro	Amuc_1675	42 T->C	14Silent	Nt2031077	C->T	Non-coding region
HG4	Amuc_1041	549_551DelCCG	Arg184Del						
B2/4	Amuc_1041	549_551DelCCG	Arg184Del						
B1	Amuc_1041	549_551DelCCG	Arg184Del	Nt806085	Ins A	Non-coding region			

^*a*^ The two deletions in the founder strain Muc^T^ were found in all sequenced strains. Strains B2 and B4 were found to harbor identical mutations, so further analysis was performed on only one of them, namely B2. Similarly, the same mutations were found in HG1 and HG2, as well as in LG1 and LG2, from which we analyzed HG1 and LG1, respectively. For further physiological and molecular analysis, strains B1, B2, LG1, HG1, HG3, and HG4 were used.

No additional mutations were found in the genomes of the six isolated and characterized strains obtained from either the control or shaking mucin conditions, compared to the founder strain Muc^T^ (Table 1). This is in line with our expectations, as we selected for the fastest grower, and *A. muciniphila*’s natural environment is mucus. Therefore, it is reasonable to conclude that the genome of *A. muciniphila* is already highly optimized for growth under these conditions.

For the Low GlcNAc condition, the two sequenced strains (LG1 and LG2) turned out to be genomically identical, with mutations found in three genes. Firstly, the loss of a nt triplet was found in the gene with locus tags Amuc_1041 resulting in the deletion of a single amino acid at position 184 in its gene product; secondly, Amuc_1412, resulting in a substitution of Gln to Pro at position 184 in its deduced protein, and finally, Amuc_1413, where a duplication in a nonanucleotide homopolymeric G tract leads to a frameshift at amino acid 55 of the predicted Amuc_1413 protein, leading to an early stop codon, reducing its size from 735 to only 98 amino acids (Table 2). For further testing, we used strain LG1. The mutation rate for *A. muciniphila* grown in Low GlcNAc medium was found to be 2.14 × 10^-10^ mutations per nucleotide per generation.

In the High GlcNAc condition, two strains (HG1 and HG2) were found to have an identical genome sequence different from the founder strain Muc^T^ (Table 2). Both strains were found to have identical mutations in the genes with locus tags Amuc_1412 and Amuc_1413, leading to a single amino acid change in the Amuc_1412 protein from Gln to Pro at position 134, and the same frameshift in the nonanucleotide homopolymeric G tract, resulting in truncation of the Amuc_1413 protein as mentioned for the Low GlcNAc condition (Table 2). For further testing, we used strain HG1. Strain HG3, which was also recovered from the High GlcNAc condition, showed three mutations: one leading to the Gln184Pro substitution in the Amuc_1412 protein, identical to that in strains HG1 and HG2; one silent nucleotide variation in Amuc_1675; and finally, a mutation in a non-coding region at nt 2031077 (Table 2). Lastly, strain HG4 showed a single mutation in the gene with locus tag Amuc_1041, leading to the deletion of a single amino acid at position 184 in the predicted Amuc_1041 protein. With this, we calculated the mutation rate for *A. muciniphila* grown in High GlcNAc medium to be 5.63 × 10^-10^ mutations per nucleotide per generation.

Two of the sequenced strains from the Bile Low GlcNAc condition, B3 and B5, did not contain any mutations (Table 1). In contrast, two other strains, B2 and B4, obtained after 1,000 or 500 generations of growth, respectively, were genetically identical, sharing a single mutation leading to the deletion of a single amino acid at position 184 of the Amuc_1041 protein, similarly to LG1 and LG2. Details on all mutations can be found in Table 2. Finally, strain B1 not only harbored the same mutation as strains B1 and B4, but also featured an A insert in a non-coding region at nt 806085. Since strain B1 was obtained after 1,000 generations, it is likely that it derived from strain B4, isolated after 500 generations, that already contained the same mutation (Table 2). Strains B1 and B2 were used for further testing. Based on the observed number of isolates and mutations, we estimated the mutation rate for *A. muciniphila* grown in Bile Low GlcNAc medium to be 8.58 × 10^-11^ mutations per nucleotide per generation.

### Generated mutants produce Amuc_1100

As the *A. muciniphila* Amuc_1100 protein is thought to be of great importance in establishing *A. muciniphila*’s potential health effects, we looked at the production of Amuc_1100 in the six selected mutant strains (Table 2) and compared it to that of the founder strain Muc^T^. For this, we measured Amuc_1100 production in pasteurized cells using western blot (Fig. S1). All six selected mutants showed a clear signal for Amuc_1100. None of the mutations are located near the gene that codes for Amuc_1100, nor are they predicted to be associated with Amuc_1100. So, we conclude that all mutants we tested still produced the Amuc_1100 protein.

### Amuc_1413 is essential for mucin binding

**Fig 2:**
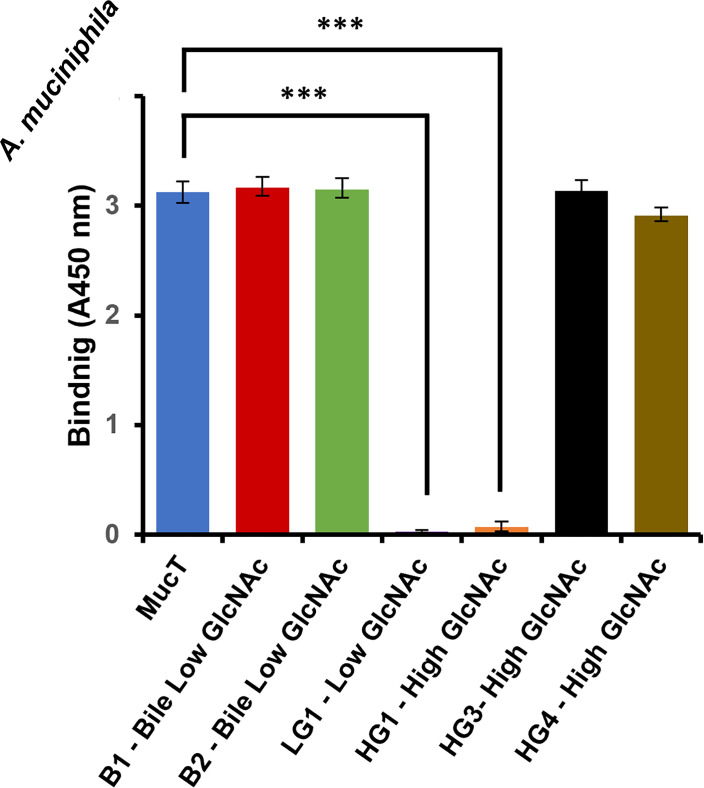
Binding of pasteurized *A. muciniphila* strains to porcine gastric hog mucin. Binding was quantified by ELISA, using absorbance at 450 nm as a readout. Bars and data points represent mean ± SD of three biological replicates. ***P < 0.001 (one-way ANOVA, Dunnett’s test).

**Fig 3:**
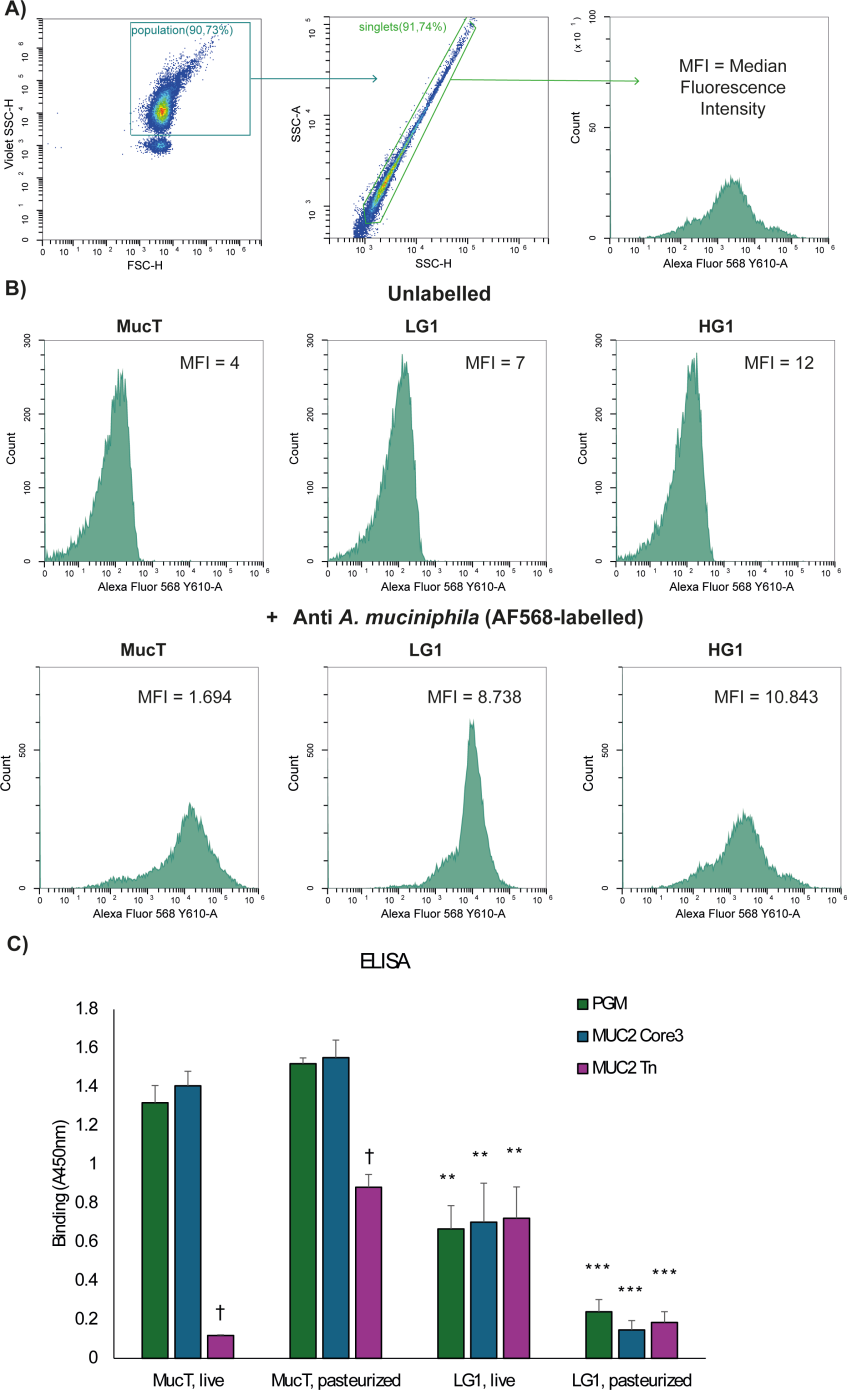
Flow cytometry analysis of Muc^T^, LG1, and HG1. (**A**) Gating scheme of *A. muciniphila*, using Muc^T^ as an example. Cells were selected based on FSC-H/Violet SSC-H. Singlets were selected based on SSC-A/SSC-H, and binding by anti-*Akkermansia* whole-cell serum was based on fluorescence in the Y610 channel (detected by Alexa Fluor (AF)568-conjugated secondary antibody). (**B**) Histograms showing distribution of fluorescence intensity in Y610 of Muc^T^, LG1, and HG1 with and without labeling with anti-*Akkermansia* whole-cell serum. Based on the median fluorescence intensity (MFI), LG1 and HG1 do not show loss of recognition by the anti-*Akkermansia* whole-cell serum. (**C**) Mucin-binding assay by ELISA using PGM (50 ng), MUC2 Core3, and Tn (12.5 ng coated) by live and pasteurized Muc^T^ and LG1. Bacteria were added at OD_600_ = 1.4, and binding was quantified using absorbance at 450 nm. Bars represent mean ± SD of three biological replicates. ** = *P* < 0.001 and *** = < 0.0001 (Student’s *t*-test, using binding of MucT to the same mucin as a reference), and † = *P* < .001 (Student’s *t*-test, using binding to Core3 by the same strain as a reference).

To determine the mucin binding capacity of the (pasteurized) mutant strains, a mucin-binding assay was performed using porcine mucin. Binding was assessed by ELISA using antiserum against whole cells of *A. muciniphila*, as done previously (27). We found that LG1 and HG1 show strongly reduced capability to bind to mucin (*P* < 0.001), similar to LG2 and HG2 (data not shown), while all other strains bound in similar amounts to Muc^T^ (Fig. 2). Flow cytometry analysis was performed to verify whether the loss of binding was not due to reduced recognition or absence of recognition by anti-whole-cell serum (Fig. 3A and B). Strain LG1 was selected to further investigate binding to more defined mucin (Tandem Repeat) O-glycodomains MUC2 Core3 and Tn (28), which revealed that live LG1 showed slightly higher binding than pasteurized cells, but still lower than Muc^T^ and without discrimination between Core3 and Tn, which was previously observed for Muc^T^ (27) (Fig. 3C).

Both LG1 and HG1 have a G insertion in the 9G homopolymer sequence in the Amuc_1413 gene, encoding a putative exopolysaccharide (EPS) biosynthesis protein, which is further explored below.

### Frameshift leads to truncation of Amuc_1413, encoding a putative EPS biosynthesis protein

The mutation occurring in the Amuc_1413 gene, observed multiple times in strains HG1, HG2, LG1, and LG2, consists of a G insertion that changes a 9G repeat to a 10G repeat, leading to a frameshift in a predicted chain length determinant protein (Wzz) domain of the protein. The first 72 amino acids of the protein are unaffected, then 26 amino acids are changed, and finally, the protein is truncated as the reading frame meets a stop codon. This leads to a reduction of the whole protein from 735 to only 98 amino acids.

Amuc_1413 is annotated as a gene coding for a capsular polysaccharide biosynthesis protein on NCBI and was previously found to be consistently highly abundant throughout growth conditions (22, 26). Proteome analysis indicated that the relative abundance of the Amuc_1413 encoded protein was similar in the type strain Muc^T^ grown in both mucin and glucose media, with a 1.3-fold change between the conditions and a log count per million reads of 9.7, placing it in the top 22% highest expressed genes in their data set (29). This suggests that the Amuc_1413 protein is always expressed in relatively large quantities.

Based on InterPro, Amuc_1413 is predicted to contain, among others, a tyrosine-protein kinase Wzc-like, C-terminal domain and a Wzz domain, which are known to be involved in EPS and/or CPS export and synthesis in other bacterial species, such as *E. coli*. In addition, Foldseek similarity search yielded similarities of the Amuc_1413 deduced protein with that of tyrosine protein kinases and (exo)polysaccharide synthesis enzymes (File S1). The most notable ones are Tyrosine-protein kinases from *E. coli*, PslE from *Pseudomonas aeruginosa*, and ExoP from *Sinorhizobium meliloti*. These are all required for (exo)polysaccharide assembly and essential for biofilm formation and surface attachment (30–33). More specifically, EPS are known to (indirectly) affect interactions of intestinal bacteria with the host environment (34) and adhesion to mucus (35). In this context, the lack of mucus binding by both LG1 and HG1 makes it likely that Amuc_1413 is indeed involved in EPS synthesis.

### Glutamine to proline in residue 134 in putative porin Amuc_1412

In strains HG1, HG2, HG3, LG1, and LG2, a mutation was detected in the Amuc_1412 gene. In its deduced protein, amino acid 134 is changed from glutamine to proline, due to a single nucleotide change of A to C, at position 401. Amino acid 134 is not part of the main outer membrane protein barrel domain of the predicted Amuc_1412 protein, but rather a part of a predicted domain of unknown function. However, it is worth noting that this domain has a relatively high E value (0.02), making it unclear if this domain holds functional significance within the protein.

Amuc_1412 is annotated as a gene coding for a hypothetical protein in NCBI, but a search in Foldseek led to high similarities with porins, such as Porin D, OpdH, and OpdK from *P. aeruginosa* PAO1, which are involved in the uptake of small molecules, basic amino acids, and tricarboxylic acids (File S1) (36–38). Previous research found Amuc_1412 to encode an outer membrane or extracellular-associated protein, fitting the description of a porin (23, 29). Its relative abundance in proteomes was found to be similar in both mucin and glucose media, with a log10 LFQ fold change of 1.9, and a log count per million reads of 10.4 placing it in the top 13% highest expressed genes in their data set (29). We used ColabFold v.1.5.5: AlphaFold2 using MMseqs2 to predict changes in AlphaFold structure and found that the overall structure, that is, the β-barrel, remains preserved. However, one β-strand bends inward 1 AA earlier, at position 134 instead of 135. This also leads to rearrangements within the barrel, indicating that the mutation might affect the protein’s functional properties (File S2). However, we did not find any differences in the growth rate, mucus binding, or metabolite production in the strains with a mutation in Amuc_1412.

### Arginine deletion in putative RNA polymerase Amuc_1041

Strains HG4, LG1, LG2, B1, B2, and B4 all carry the same mutation in the gene Amuc_1041, which is annotated in the NCBI database as coding for the DNA-directed RNA polymerase subunit beta (RpoB). The mutation involves a deletion of a CCG codon at positions 549–551, reducing a repeat of six CCG codons to five. As a result, arginine at position 184 of the 1,312-amino acid protein is deleted. We used ColabFold v.1.5.5: AlphaFold2 using MMseqs2 to predict changes in AlphaFold structure and found that predicted structure of the protein was unaffected by the deletion of arginine at position 184 (File S3).

The Amuc_1041 protein was previously found to be expressed and cytoplasm-associated in proteomic analyses (26). We did not find any differences in the growth rate, mucus binding, or metabolite production in strains with a mutation in Amuc_1041. The mutation in the Amuc_1041 gene occurred independently in three of the five conditions and consists of a sixfold repetitive trimer, one of which is lost.

It is possible that this triple deletion is a result of slipped-strand mispairing, in which case a different number of nucleotide repeats can be found (39). Often, this leads to a specific phenotype, but in this case, in *A. muciniphila,* we did not find this and hence assume this is likely to be a silent mutation.

### Silent mutations and non-coding regions

The mutation found in strain HG3 in the Amuc_1675 gene is silent, involving a T to a C transition at position 42, located in a non-coding area of the gene. Finally, we also encountered two mutations in non-coding regions of the *A. muciniphila* genome. One is the mutation found in strain B1, which is an insertion of A at nucleotide 806085, located in the non-coding region between Amuc_0685 and Amuc_0686. The other is found in strain HG3 and is a nucleotide change from C to T at position 2031077, located in the non-coding region between Amuc_1675 and Amuc_1676. We did not observe any effect of these mutations on the growth rate, mucus binding, or metabolite production.

## DISCUSSION

In this study, we examined the long-term genomic stability of *A. muciniphila* Muc^T^ after growing for more than 1,000 generations under five different culturing conditions. We isolated individual colonies from each condition and sequenced their genomes to identify mutations and assess the strains’ genomic stability.

We found that *A. muciniphila* Muc^T^ exhibited remarkable stability, particularly when grown in mucin. After 1,000 generations, no detectable mutations were found in strains grown on mucin. Similarly, the *A. muciniphila* genome was found to be highly stable when grown in medium supplemented with Glc and GlcNAc, with mutation rates between 5.63 × 10^−10^ and 2.14 × 10^−10^ mutations per nucleotide per generation. The mutation rate when *A. muciniphila* was grown in medium with added bile was 8.6 × 10^−11^ mutations per nucleotide per generation. This may be a consequence of the bile stress, since this observed rate is slightly lower than the reported spontaneous mutation rate for *E. coli*, which ranges between 0.2 × 10^−10^ and 5.4 × 10^−10^ mutations per nucleotide per generation (40, 41). Interestingly, *A. muciniphila* appears to be considerably more stable than *Lacticaseibacillus rhamnosus* GG, which is widely marketed as a probiotic. This is because *L. rhamnosus* GG suffers from deletions through IS-element-mediated recombinations, leading to a higher mutation rate of between 2.65 × 10^−9^ and 3.98 × 10^−9^ mutations per nucleotide per generation (42).

However, it is important to note that our mutation rate estimates might be conservative, as we sequenced the genomes of only 18 strains selected based on potential phenotypic deviations from the type strain. Many mutations would not lead to a different phenotype and would have been missed in our analysis. Despite this limitation, it is still remarkable that no mutations were found in the genomes of any of the seven sequenced strains grown on mucin. This may be explained by the fact that mucus degradation is a complex process involving a host of genes that are conserved in the genomes of *Akkermansia* spp (12), which likely reduces the survival of any mutants that are affected in these genes. In addition, the natural environment of *A. muciniphila* is mucus, so it is likely that this symbiont is most optimized for living in mucus, and thus any mutation would be rarely beneficial.

It is remarkable that several of the same mutations arose independently across different conditions and growth experiments, suggesting the possibility of mutational hotspots (43). For example, the Amuc_1041 gene, which encodes a putative RNA polymerase, exhibited the same mutation, involving a reduction of a six-times CCG repeat to five, across different conditions. We did not find any differences between the strains with a mutation in Amuc_1041 and the founder strain Muc^T^ in either growth rate, mucus binding, or metabolite production.

Another hotspot that was revealed in our screening included the Amuc_1413 gene, encoding a putative EPS biosynthesis protein. In four of the nine sequenced mutants, a mutation occurred in this gene, turning a homopolymeric G stretch from 9 to 10 G residues at position 164. This insertion results in a frameshift mutation, truncating the Amuc_1413 protein from its full length of 735 to 98 amino acids. Functional analysis of two of the mutants revealed a significant reduction in their ability to bind mucin. Given the early truncation of the protein, it is likely that the function of the Amuc_1413 protein is completely disrupted, resulting in loss of mucus-binding capacity in these strains. Altogether, this leads us to conclude that Amuc_1413 is essential for mucus binding.

Interestingly, the Amuc_1413 frameshift that was found during sequencing was found in between 87% and 91% of the reads across all samples, while all other mutations, like those in Amuc_1401, exhibited rates between 98% and 100%. This suggests that the mutation in Amuc_1413 is dynamic, potentially indicating a form of phase variation. Phase variation is characterized by reversible mutations, often frameshifts, enabling a bacterial population to rapidly switch gene expression on and off to adapt to changes in the environment. Such variation is often associated with sequences prone to slippage, like poly G-tracts, with nearby frameshifted stop codons (44). This is exactly what we observed here in the case of Amuc_1413, as this gene is surrounded by several out-of-phase stop codons. This type of variation has been reported in pathogens but also in gut-associated bacteria, such as *Bifidobacterium breve* UCC2003, where pilus expression is heterogeneous within a population, regulated by two poly G-tracts in pili-related gene clusters. In *B. breve* UCC2003, the population is enriched for “ON” sequences in these genes after passage through a mouse model, suggesting that pili have functional advantage in the mouse gut, likely aiding retention (45). It is likely that the putative EPS biosynthesis protein, Amuc_1413, has a similar regulatory mechanism, where its nonanucleotide homopolymeric G tract mediates phase variation. We found that Amuc_1413 is essential for mucus binding, and the frameshift mutant was found only in samples originating from media without mucus. In the absence of mucus, there is no selective advantage for maintaining mucus-binding capability. Given the high metabolic cost associated with EPS production, the phase variation in Amuc_1413 may favor a truncated, non-functional form of its encoded protein in these mucus-free conditions.

To explore whether this potential phase variation in Amuc_1413 is a conserved feature across *Akkermansia* species, we analyzed 100 genomes of *Akkermansia* spp. deposited in the NCBI database, for the presence and length of the homopolymer G tracts in Amuc_1413 by using NCBI Nucleotide Blast (accessed on 24 September 2024) (Table S3). We found 32 genomes that have the 9G homopolymer tract, like the reference strain *A. muciniphila* Muc^T^, suggesting these may also undergo phase variation. Additionally, four other genomes could also undergo phase variation, three of which have an 8G tract, while one has a 10G tract. All these variations result in an Amuc_1413 gene that is truncated after amino acid 79 (8G) and 98 (10G), compared to the 735-aa full protein. This means that it is unlikely that Amuc_1413 was functional at the time of sequencing these genomes.

The observed loss of mucus binding capacity in *A. muciniphila* strains with a truncated Amuc_1413 protein with a predicted role in EPS transport is compatible with the known involvement of EPS in adherence and biofilm formation (34). Moreover, our findings on Amuc_1413 confirm and extend previous research by Davey et al. who demonstrated that disruption of this gene after transposon mutagenesis led to a strain with reduced capabilities to colonize the mouse gastrointestinal tract (11), which could be a consequence of reduced mucus binding. However, it is important to note that transposon insertion can cause polar effects by disrupting expression of downstream genes, often affecting entire operons or gene clusters. In contrast, here we show similar results in reduced binding to mucus in mutants with only a point mutation in Amuc_1413, leading to disruption of this gene only. Our previous work showed that binding of *A. muciniphila* to mucus is O-glycan-specific, but the binding ligand remains to be identified (27). To our knowledge, EPS are known to be mainly involved in primary (non-specific) adhesion to mucus (35, 46). EPS have also been found to impact pili formation in several ways (47, 48), further strengthening the generally suspected role of the type IV pili of *A. muciniphila* in adhesion and signaling (9).

This work has a limited number of weaknesses. One is that we used Illumina-based re-sequencing of the genomes to identify deviations with the *A. muciniphila* Muc^T^ closed genome sequence that was determined using first- and second-generation sequencing methods. While no single-molecule sequencing was used, we are confident that we captured most of the diversity as our analysis was identical to that of Davey et al. (11) in detecting a single nucleotide mutation in the published *A. muciniphila* MucT gene sequence (25). Another one is that we isolated a limited number of cells after subculturing for 500 and 1,000 generations for simple practical and budget reasons. Nevertheless, we did observe readily the phase variation in the cells that were grown in media devoid of mucus. Finally, while we identified Amuc_1413 as a crucial player in mucus binding, we did not yet explore the mechanism by which it affects binding to mucus. This would greatly benefit from a host-vector system to generate defined mutations, but this has yet to be developed for *A. muciniphila*.

Our results show a remarkable stability of the *A. muciniphila* genome when cultivated in mucin medium. However, for research and industrial applications, defined media are to be preferred as mucus often contains impurities, interferes with downstream processing, and is incompatible with food or pharmaceutical application. Our research shows that growing continuously in an industry-like synthetic medium supplemented with Glc and GlcNAc is a good alternative, resulting in a minimal number of mutations, even after 1,000 generations. However, it remains crucial to regularly monitor mutations, as for large-scale industrial productions at different sites worldwide using different cell banks, the number of generations can be considerable. Specifically, it is important to consider the stability of the homopolymer G tract in Amuc_1413, coding for a putative EPS biosynthesis protein. A single mutation in the Amuc_1413 gene resulted in the loss of mucin binding, which might be very relevant to the functional properties of *A. muciniphila*; it is crucial for its persistence within the human GI tract.

## MATERIALS AND METHODS

### Growth conditions and set-up

*Akkermansia muciniphila* Muc^T^, obtained from the Wageningen Microbiology Culture Collection, was cultured at 37°C in 30 mL MM as described previously, in five different conditions (2, 23). For the control condition, 0.5% porcine gastric mucin (PGM, type III, Sigma-Aldrich, USA) was added to the MM. The shaking condition consisted of the same medium as the mucin control, with the addition of continuous shaking at 200 rpm. The high GlcNAc condition contained 4.5 g/L Glc and 5.5 g/L GlcNAc, an equimolar ratio. The low GlcNAc condition contained 8.1 g/L Glc and 1.1 g/L GlcNAc, 10:1 molar ratio. Finally, the bile-induced stress condition consisted of the same medium as the low GlcNAc condition, with the addition of 0.1% (wt/vol) ox bile (ox bile for microbiology, Sigma-Aldrich, USA). All conditions containing Glc and GlcNAc were supplemented with 20 g/L tryptone and 4 g/L L-Threonine. Culturing was performed in serum bottles sealed with butyl rubber stoppers under anaerobic conditions with 1.5 atm N_2_/CO_2_.

The generation time of *A. muciniphila* was determined for each condition (between 13 and 14 generations in 24 hours) (Table S1). After incubating for 24 hours, cells were still growing exponentially, as evidenced by their OD. Approximately 1,000 generations were cultured by diluting each sample 1,000 times daily in fresh culture medium for 100 consecutive days. Purity of the cultures was regularly analyzed by plating and checking morphology of the colonies, microscopy, and weekly 16S rRNA amplicon sequencing.

At both generation 500 and 1,000, cultures of each condition were plated on brain heart infusion plates with 0.5% PGM (BHI+ plates) and were grown at 37°C for 2 days in anaerobic jars. From each plate, seven colonies were randomly picked and re-streaked and grown again for 2 days at 37°C on BHI+ plates. Colonies were plate-purified, and from these plates, one colony was picked and grown at 37°C overnight in the high GlcNAc medium. The strains were stored in 25% glycerol, 25% phosphate-buffered saline (PBS) at −70°C.

### Metabolite quantification

Isolated strains were grown overnight in high GlcNAc medium at 37°C and subsequently, metabolites were quantified using high-performance liquid chromatography analysis as described previously (49).

### Growth curves

To generate growth curves, precultures of each strain were grown overnight at 37°C from glycerol stock in the medium used the strains were initially cultured in. Then, samples were normalized to OD_600_ = 2.8 and inoculated at 1% vol/vol in each of the five media described above, which were all flushed with CO_2_ beforehand to overcome the lack of CO_2_ in the anaerobic tent. Strains were grown in 96-well plates for 24 to 48 hours in an anaerobic tent containing 99% N_2_ and 1% H_2_. Wells were covered with mineral oil to avoid evaporation, and OD_600_ measurements were taken every 15 minutes in 96-well plates on Plate Reader ELx808 (BioTek, USA). Reads were analyzed using imager software v.3.10 (BioTek, USA) and Microsoft Excel.

### Genome analysis

Selected strains were grown overnight in high GlcNAc medium at 37°C. DNA was subsequently isolated using the MasterPure Gram-Positive DNA Purification Kit (Lucigen, USA) according to manufacturer’s protocol. Ready-Lyse incubation time was 30 minutes. Samples were sequenced using Eurofins INVIEW resequencing on Illumina NovoSeq 6000, 150 bp, PE mode, HiSeq with 2 × 150 bp paired-end reads, resulting in over approximately 5 million read pairs and minimally 100× average coverage. Sequences were cleaned and normalized, and high-quality reads were used for assembly using SPAdes (St. Petersburg genome assembler). Assembly and annotation were performed by Eurofins (the Netherlands). All samples had 19 scaffolds, a total mapped percentage between 99.92% and 99.98%, and an average coverage depth between 135 and 147. The received paired-end genomes were mapped against the genome of the reference strain *A. muciniphila* Muc^T^ and analyzed for mutations using PATRIC and Geneious 10.0.9 (25, 50). In PATRIC, a Variation Analysis was performed using aligner BWA-MEM and SNP caller FreeBayes. In Geneious, we used the medium sensitivity mapper setting. The nucleotide coverage varied between 66- and 2,189-fold (Table S2), and nucleotides with a coverage below 100 were checked visually for variations using Geneious. The founder strain used in this study was obtained from a glycerol stock of the original *A. muciniphila* Muc^T^ from the Wageningen Microbiology Culture Collection.

### Mutation analysis

To get an impression of possible functionalities of the affected genes, we first looked at potential functionality as annotated in NCBI by BlastP. The full sequence was run against RefSeq Select Proteins database using blastp (protein-protein BLAST) and default parameters. Then, we used Foldseek to compare their predicted structure and protein sequence by AlphaFold to several databases ([51]; Mode 3Di/AA, non-iterative search, default parameters), and STRING to look at predicted functional partners ([52]; network type: full STRING network; interaction sources: text mining, experiments, databases, co-expression, neighborhood, gene fusion, and co-occurrence; minimum required interaction score: 0.400). In addition, we checked domain functionalities using InterPro (53). We used the KEGG sequence similarity database motif to determine in which motif the mutations occur, and ColabFold to visualize the effect of the mutation on the predicted structure by AlphaFold, whenever applicable (54, 55).

### Pasteurization

All selected strains were cultured in high GlcNAc medium at 37°C for 24 hours for 5 consecutive days, subculturing daily, to reduce any potential variation caused by the different media they were originally cultured in. After that, samples were centrifuged for 15 minutes at 15,000 *g*, supernatant was removed, and samples were concentrated in PBS. Then, samples were pasteurized by incubation for 30 minutes at 70°C. After pasteurization, samples were normalized to OD_600_ = 1.0 and stored at −20°C until further use. All pasteurized samples were plated on BHI+ plates and incubated anaerobically at 37°C for 2 days to ensure inactivation.

### Amuc_1100 protein production whole-cell antibody recognition

To determine the production of the pili-associated signaling protein Amuc_1100, Western blot analysis was performed. Pasteurized samples were heated for 10 minutes at 95°C in Laemmli buffer and loaded on 4–15% Novex Tris-Glycine Mini Protein Gels, 4–20% (Thermo Fisher Scientific, Eindhoven, The Netherlands). Proteins were transferred onto a polyvinylidene difluoride membrane using the iBlot 2 Dry Blotting System (Thermo Fisher Scientific). Membranes were blocked for 1 hour and incubated overnight at 4°C with the primary antibody, rabbit anti-Amuc_1100 (Eurogentec, Belgium). The blocking solution was PBS with 0.1% Tween 20 and 3% bovine serum albumin, which was also used to prepare the antibody solutions. Next, membranes were incubated for 1 hour with goat anti-rabbit antibody (Thermo Fisher Scientific). Signals were quantified using the ChemiDoc MP system (Bio-Rad, Lunteren, The Netherlands) and SuperSignal West Pico PLUS substrate rabbit (Thermo Fisher Scientific).

### Mucus binding assay

Mucus binding was assessed by ELISA as described previously (27). MaxiSorp 96-well plates (Nunc) were coated with 50 µL 0.1% PGM in carbonate-bicarbonate buffer (pH 9.6), shaking at 250 rpm, 4°C overnight. Plates were blocked with 100 µL PLI-P (0.5 M NaCl, 3 mM KCl, 1.5 mM KH_2_PO_4_, 6.5 mM Na_2_HPO_4_ · 2 H_2_O, 1% Triton X-100, 1% bovine serum albumin (BSA), pH 7.4) for 1 hour at RT and incubated with pasteurized bacteria (OD_600_ = 1.2). Plates were incubated with anti-*Akkermansia muciniphila* (whole-cell) serum (prepared as described previously [9], 1:1,000 in PLI-P) for 1 hour at 4°C, followed by incubation with 1 µg/mL HRP-conjugated polyclonal goat anti-rabbit IgG (H+L) (Invitrogen) for 1 hour. In between incubations, plates were extensively washed with PBS-T (PBS containing 0.05% Tween-20). The binding was detected by addition of TMB substrate (ThermoFisher Scientific) and stopped with an equal volume of 0.5 M H_2_SO_4_. The absorbance at 450 nm was measured (Agilent BioTek, Gen5 Software). Dunnett’s multiple comparisons tests were used to determine statistical significance. To further investigate the binding of Muc^T^ and LG1, plates were coated with 0.0001% PGM or 250 ng/mL mucin reporters MUC2 Core3 and Tn (28) and binding of both live and pasteurized bacteria was tested at OD_600_ = 1.4.

### Flow cytometry analysis

Muc^T^ and LG1 were grown overnight in basal medium supplemented with 20 g/L tryptone and 4 g/L L-Threonine, 0.25% (wt/vol) Glc and 0.275% (wt/vol) GlcNAc. Cells were washed in filter-sterile PBS and resuspended at OD_600_ = 0.28 in PBS supplemented with 4% paraformaldehyde to fix in a final volume of 150 µL. After fixation for 20 minutes, cells were washed three times in PBS and blocked in PBA (PBS with 1% BSA) for at least 30 minutes. Next, cells were incubated for 1 hour with anti-*Akkermansia muciniphila* (whole-cell) serum at 4°C, washed three times in PBS, and incubated with Alexa Fluor 568 goat anti-rabbit IgG (H+L) (Thermo Fisher, A-11011) at 4°C. After washing, cell pellets were finally resuspended in 0.5 mL of PBS and analyzed using the CytoFLEX S (Beckman Coulter, Inc.). Data were analyzed using the CytExpert software version 2.6 (Beckman Coulter, Inc.).

## Data Availability

The genome sequences of all *A. muciniphila* derivatives were deposited in NCBI’s Sequence Read Archive (SRA). The BioProject accession number is PRJNA1152743.

## SUPPLEMENTAL MATERIAL

File S1 spectrum.02400-25-s0001.xlsx Foldseek results.

File S2 spectrum.02400-25-s0002.txt Amuc1412MT Colabfold Log.

File S3 spectrum.02400-25-s0003.txt Amuc1041MT Colabfold Log.

File 4 spectrum.02400-25-s0004.xlsx VCF.

Figure S1 spectrum.02400-25-s0005.tif Amuc_1100 protein production.

Supplemental material spectrum.02400-25-s0006.docx Tables S1 to S3 and Legends for Files S1 to S3 and Figure S1.

ASM does not own the copyrights to Supplemental Material that may be linked to, or accessed through, an article. The authors have granted ASM a non-exclusive, world-wide license to publish the Supplemental Material files. Please contact the corresponding author directly for reuse.
