# Supplementary figures and images for: High stability of the genome of Akkermansia muciniphila MucT under long-term culturing conditions
Source: Microbiol Spectr. 2026 Mar 10;14(4):e02400-25. doi: 10.1128/spectrum.02400-25 (PMC13055324; doi:10.1128/spectrum.02400-25)

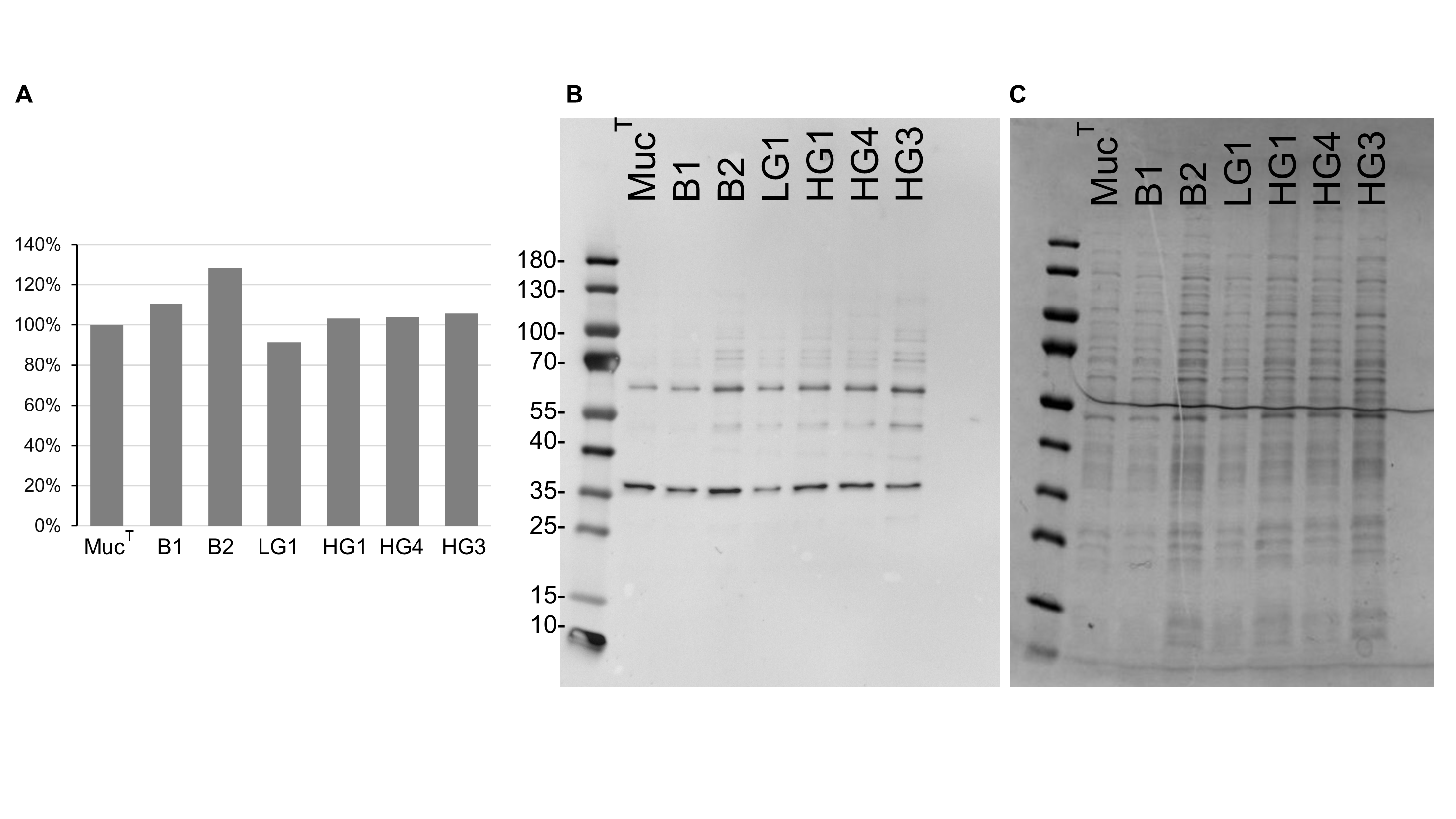

Supplement: Figure S1 — Amuc_1100 protein production. [file spectrum.02400-25-s0005.tif]
