# Supplementary material for: High stability of the genome of Akkermansia muciniphila MucT under long-term culturing conditions
Source: Microbiol Spectr. 2026 Mar 10;14(4):e02400-25. doi: 10.1128/spectrum.02400-25 (PMC13055324; doi:10.1128/spectrum.02400-25)
Supplement: Supplemental material — Tables S1 to S3 and Legends for Files S1 to S3 and Figure S1. [file spectrum.02400-25-s0006.docx]

Supplementary Material

**Supplementary File 1: Foldseek results**

**Supplementary File 2: ColabFold log file for Amuc_1412 mutant**: Glutamine to proline in residue 134.

**Supplementary File 3: ColabFold log file for Amuc_1041 mutant:** Arginine 184 deletion.

**Supplementary File 4: VCF files**

Supplementary Figure Legends

**Supplementary Figure 1. Amuc_1100 protein production** A) Normalized Amuc_1100 production corrected for total protein, B) Western blot with anti-Amuc_1100 antibodies, C) Coomassie total protein gel staining.

Supplementary Tables

**Supplementary Table 1: Number of generations per day based on CFU**

|  | Colonies at 24h (dil -2) | CFU/ml inoculation | Colonies at inoculation (dil. -5) | CFU/ml 24h | # gen |
| --- | --- | --- | --- | --- | --- |
| Control mucin | 2 | 2.00E+04 | 38 | 3.80E+08 | 14.2 |
| Shaking mucin | 3 | 3.00E+04 | 30 | 3.00E+08 | 13.3 |
| Low GlcNAc | 3 | 3.00E+04 | 30 | 3.00E+08 | 13.3 |
| High GlcNAc | 10 | 1.00E+05 | 142 | 1.42E+09 | 13.8 |
| Bile Low GlcNAc | 6 | 6.00E+04 | 49 | 4.90E+08 | 13.0 |

**Supplementary Table 2: Highest and lowest nucleotide coverage per sequenced strain**

|  | # of reads at lowest coverage nt | # of reads at 2nd lowest coverage nt | # of reads at highest coverage nt |
| --- | --- | --- | --- |
| B1 | 89 | 206 | 1319 |
| B2 | 109 | 230 | 1550 |
| B3 | 113 | 275 | 1525 |
| B4 | 100 | 220 | 1763 |
| B5 | 101 | 298 | 1830 |
| HG1 | 96 | 241 | 1408 |
| HG2 | 93 | 259 | 1740 |
| HG3 | 107 | 270 | 1482 |
| HG4 | 83 | 293 | 1320 |
| LG1 | 128 | 226 | 1262 |
| LG2 | 101 | 216 | 1847 |
| M1 | 66 | 235 | 1704 |
| M2 | 141 | 361 | 2607 |
| MucT | 91 | 278 | 1723 |
| S1 | 88 | 190 | 1443 |
| S2 | 98 | 257 | 1630 |
| S3 | 116 | 333 | 2000 |
| S4 | 75 | 186 | 1774 |
| S5 | 74 | 319 | 2189 |

**Supplementary Table 3. Variations in the homopolymer tract of Amuc_1413 in 100 *Akkermansia* strains.** Data obtained from NCBI Nucleotide blast (accessed on 24-09-2024).

| Amuc_1413 homopolymer tract variation | Occurrence | Example strains |
| --- | --- | --- |
| GGGGGGGGGG | 1 | MnAkk1 |
| GGGGGGGGG | 32 | Muc^T^, ATCC-BAA-835 |
| GGGGGGGG | 3 | AMDK-3, AMDK-24, AMDK-25 |
| GGGGAGGGG | 23 | H2 |
| GAGGGGGGG | 34 | Akk1756, Akk0500b |
| AGGGGGGGG | 6 | All *Akkermansia massiliensis* |
| GAGGGGGG | 1 | NB2A-8-WC |
